# Educating women about congenital cytomegalovirus: assessment of health education materials through a web-based survey

**DOI:** 10.1186/s12905-014-0144-3

**Published:** 2014-11-30

**Authors:** Simani M Price, Erika Bonilla, Paul Zador, Denise M Levis, Christina L Kilgo, Michael J Cannon

**Affiliations:** Westat, Inc., 1600 Research Blvd., Rockville, MD 20850 USA; National Center on Birth Defects and Developmental Disabilities, Centers for Disease Control and Prevention (CDC), 1600 Clifton Road, Mailstop E-86, Atlanta, GA 30333 USA; Carter Consulting, Inc., 2310 Parklake Drive NE, Suite 535, Atlanta, GA 30345 USA

**Keywords:** Cytomegalovirus, Congenital, Health education materials, Web-based survey, Prevention guidelines, Pregnant women

## Abstract

**Background:**

Congenital cytomegalovirus (CMV) is the most common congenital infection in the U.S. and can result in permanent disabilities, such as hearing and vision loss, intellectual disability, and psychomotor and language impairments. Women can adopt prevention behaviors in an attempt to reduce their exposure to CMV. Currently, few women are familiar with CMV. To increase awareness of CMV, the Centers for Disease Control and Prevention (CDC) developed draft health education materials. The purpose of this study was to pilot test two health education materials to gauge their appeal and to determine if they increase knowledge about CMV and motivate audiences to seek additional information on CMV and adopt CMV prevention behaviors.

**Methods:**

African-American (n = 404) and Caucasian women (n = 405), who had a young child and were either pregnant or planning a pregnancy, were recruited to participate in a 15-minute web survey. Participants were randomly assigned to view one of two CMV health education materials, either a factsheet or video. Pre and post survey measures were used to assess changes in knowledge of CMV and motivation to adopt prevention behaviors. We also examined audience preferences regarding materials and motivation.

**Results:**

CMV knowledge score increased significantly after presentation of either the video or factsheet (from 3.7 out of 10 to 9.1 out of 10, p <0.001). The average materials appeal score was high, with a mean of 3.6 on a four-point scale, indicating women responded very positively to both materials. Regression analyses indicated that appeal, message involvement (e.g., information seeking, discussing with others), post materials knowledge score, and viewing the video (vs. factsheet) were significantly positively associated with increased support for CMV prevention behaviors.

**Conclusions:**

Overall, we found that the health education materials improved women’s knowledge of CMV and encouraged them to adopt prevention behaviors. Given the low awareness levels among women currently, these findings suggest that appropriate education materials have the potential to greatly increase knowledge of CMV.

As women become more knowledgeable about CMV and transmission routes, we expect they will be more likely to adopt prevention behaviors, thereby reducing their risk of CMV infection.

**Electronic supplementary material:**

The online version of this article (doi:10.1186/s12905-014-0144-3) contains supplementary material, which is available to authorized users.

## Background

An estimated 28,000 infants are born each year with congenital cytomegalovirus (CMV), the most common congenital infection in the U.S.; of those infected, approximately 150-200 children will die and nearly 20% will suffer permanent disabilities, such as hearing and vision loss, intellectual disability, psychomotor delays, and speech and language impairments [1–3].

Congenital CMV infection is a result of intrauterine CMV transmission from mother to fetus and can occur when a pregnant woman contracts a primary CMV infection just before or during pregnancy [4]. Congenital CMV infection can also occur when a pregnant woman has a CMV reactivation or is re-infected with a different strain of the virus [5,6]. A key source of maternal infection during pregnancy is young children (e.g., toddlers), who may shed CMV in urine and saliva [7,8]. A pregnant woman could become infected if her eyes, nose, or mouth comes into contact with an infected child’s saliva or urine [7–10].

To decrease transmission of CMV, the American Academy of Pediatrics has advised hand hygiene when caring for children, particularly after changing diapers [11]. Similarly, the American College of Obstetricians and Gynecologists has advised women with young children to use safe-handling techniques after handling diapers or after exposure to respiratory secretions [12]. In addition to this advice, researchers who have conducted CMV behavioral interventions have also advised women to avoid kissing young children on the mouth, to refrain from sharing food, drink, and utensils, and to cleanse toys and other objects that may be exposed to children’s body fluids [13,14]. However, surveys estimate that only between 14% and 22% of U.S. women have heard of CMV, and few are familiar with outcomes, transmission modes, or prevention strategies [15,16].

Currently, there is no vaccine to prevent CMV infection [17–19], routine newborn screening has not been recommended, and treatment options are limited [20–22]. Therefore, one promising approach to reduce congenital CMV infections is to educate women of reproductive age about the virus and behaviors that could reduce exposures to CMV [23]. Behaviors that limit exposures to CMV could potentially benefit both seronegative and seropositive women. To this end, the Centers for Disease Control and Prevention (CDC) is developing audience-tested, evidence-informed health education materials to motivate women to follow behaviors that could reduce exposures to CMV.

The main goals of this study were to assess CMV health education materials to see if they are appealing to women, if they increase knowledge about CMV, and if they motivate women to seek additional information on CMV and follow CMV prevention behaviors. A secondary goal was to determine if responses to materials differed by racial group because CMV prevalence and congenital CMV birth prevalence are significantly higher among non-Hispanic black persons than among non-Hispanic white persons [3,24].

## Methods

### Sampling and screening

Women were recruited using a pre-existing, non-probability web panel from Harris Interactive (HI). Quota-based sampling was used to reach a target sample of 800 with equal proportions of non-Hispanic black and white women. HI has approximately two million fully opted-in and active members in their global panel with a membership base concentrated in North America and Western Europe. To achieve the quota requirements for the non-Hispanic black sample in this study, HI also recruited participants from other panel vendors. Panel members receive incentives in the form of points from the survey provider for their participation in studies.

Participants were initially identified using pre-targeted variables based on known demographics of panel members. Email messages that included a link to the screening instrument were sent to a random sample of panel participants in batches until quota requirements were met. Of the 1,508 panel participants that completed the screening instrument, 699 did not meet the eligibility requirements for the study and 809 qualified and completed the survey.

The criteria for participation in the web survey included being a non-Hispanic black or white woman, 18-40 years of age, who was pregnant or planning a pregnancy and also had a child <5 years of age. Efforts were made to recruit a mix of educational and income levels within each racial group. Women were screened out of the survey if they had a child with a previously diagnosed disability, ever worked as a health care provider, or if their computer did not have Adobe Flash Player, which was required for viewing the health education materials.

### Study design

We used a 2 × 2 factorial design, with non-Hispanic black (n = 404) and white (n = 405) women randomly assigned to view one of two CMV health education materials, either a factsheet (n = 404) or video (n = 405). Study participants completed a 15-minute web survey that included both pre and post CMV knowledge measures.

### CMV health education materials

Findings from previously conducted focus groups of similar audiences provided guidance on effective message frames for communicating about CMV and helped refine the health education materials used in the study. The one-page factsheet was presented in a question-and-answer format that addressed three topics: what is congenital CMV, how do pregnant women acquire the infection, and how can pregnant women reduce their risk of acquiring it. The factsheet was segmented into sections by topic area, using color and white space to separate each section. The factsheet included pictures of non-Hispanic black and white children and a diagram illustrating how CMV is transmitted.

The video began with a first-person story of a non-Hispanic black mother whose child was born with CMV. This was followed by a segment explaining what health consequences CMV can cause in newborns, how common infection is, how CMV is transmitted to mothers and fetuses, which women are most at risk, and how they can reduce their risk of acquiring CMV. The video concluded with a physician summarizing the key CMV prevention behaviors. The video lasted approximately five minutes.

### Survey development and data collection

Prior to administering the survey to the full sample, cognitive interviews and pilot testing of the web survey were conducted with a small sample of the target audience to assess comprehension of survey questions, obtain feedback on the appropriateness and clarity of certain words and response categories, and gauge timing for overall survey length.

The Institutional Review Board for Westat, the contractor who handled data collection, approved the study. As part of the introduction, participants were asked to read the consent screen and click “continue” to access the survey, thereby acknowledging their agreement to participate in the survey. Participants received points from the survey company as incentive for completing the survey.

Baseline measures of risk behaviors, awareness, and knowledge related to CMV were asked of all participants prior to viewing either the factsheet or video. After completing the baseline component of the survey, participants were provided with one of the two materials at random. They were required to indicate if they could view the material on their screens before beginning the next section of the survey. Following the presentation of materials, participants were asked a series of questions designed to assess how their knowledge of CMV was affected after viewing the material (identical to baseline questions), the appeal of the health education materials, and their motivation to seek additional information on CMV and adopt CMV prevention behaviors. At the end of the survey, they were asked to assess the effectiveness of various channels for disseminating CMV information. The entire survey (see Additional file 1) took approximately 15 minutes on average to complete.

### Measures

#### Participant demographics

Women were asked to indicate their race/ethnicity, educational level, household income, and pregnancy status (currently pregnant or planning a pregnancy in the next 12 months).

#### Baseline CMV risk and protective behaviors

Using a five-point scale (1 = Never to 5 = Always), women were asked how frequently they washed their hands or used hand sanitizer after various behaviors that could expose them to their child’s urine and saliva. Using a different five-point scale (1 = Never to 5 = Everyday), women were also asked how frequently they practiced behaviors that could expose them to their children’s saliva. These survey items have been used in a previous survey [16].

#### Awareness of CMV

Using a three-point scale (1 = Very familiar, 2 = Somewhat familiar, and 3 = Not very familiar), women were asked to indicate their familiarity with several different health conditions affecting children (e.g., Down syndrome, autism, congenital CMV, etc.). The CMV familiarity rating served as an overall awareness measure of CMV.

#### Pre and post knowledge score

A series of 12 questions assessed CMV knowledge before and immediately after the presentation of the health education material. Eleven of the 12 knowledge questions had true, false, and don’t know as response options. For one question, multiple choices were presented as response categories. Correct responses to the knowledge items were summed to create a pre knowledge score (chronbach alpha = .81) and a post knowledge score (chronbach alpha = .72), with a maximum of 12 possible correct for each score.

#### Materials appeal score

Using a four-point agreement scale (1 = Strongly disagree to 4 = Strongly agree), women were asked to indicate their agreement with four statements that assessed the appeal of the CMV health education material. Responses were coded such that higher agreement with statements indicated positive reactions to the materials. One statement was given to all women (*If I were pregnant I would be worried about CMV*). Three statements were specific to the factsheet and were averaged (chronbach alpha = .76) to create a global materials appeal score for the factsheet; similarly, three statements were specific to the video and were averaged (chronbach alpha = .93) to create a global materials appeal score for the video.

#### Message involvement score

Two questions gauged participants’ reactions following exposure to the health education materials. Using a three-point Likert scale (1 = Very likely, 2 = Somewhat likely 3 = Not at all likely), participants were asked about their likelihood of looking for CMV information *(e.g., talk to your doctor or search the Internet*) or talking about CMV with their friends or family in the next week or so. The two questions were averaged (chronbach alpha = .83) to create a global measure of message involvement. Research suggests that behaviors such as searching for additional information and discussing with others are indicative of cognitive elaboration of messages and associated with increased message involvement [25].

#### Support for CMV prevention behaviors score

Using a five-point agreement scale (1 = Strongly disagree to 5 = Strongly agree), women were asked to indicate their agreement with six statements regarding whether viewing materials would encourage them to engage in CMV prevention behaviors if they were pregnant. Responses to the six items were coded such that higher agreement ratings indicated increased support for CMV prevention behaviors. The six items were combined to create an average measure (chronbach alpha = .94) of support for CMV prevention behaviors.

#### Communication channels for CMV information

Using a three-point scale (1 = Not at all effective, 2 = Somewhat effective, and 3 = Very effective), women were asked to indicate the effectiveness of various communication channels for disseminating CMV information to mothers.

### Data analysis

All analysis was conducted using SPSS 20.0 (SPSS, Inc., Chicago, IL, December 2011). Paired t-tests were used to examine differences between pre and post knowledge scores. Chi-square analysis was conducted to examine demographic differences, frequency of CMV-associated risk behaviors at baseline, and CMV familiarity rating. Analysis of variance was conducted to examine differences in knowledge scores, materials appeal, and message involvement by race and material type. In all the analyses of variance, education, income, and pregnancy status were included in the model as covariates. For the analysis of post knowledge scores, pre knowledge score was also included as a covariate.

We conducted multiple linear regression to examine the association between the support for CMV prevention behavior score (dependent variable) and several predictor variables (i.e., post knowledge score, materials appeal, message involvement, material type, and participant demographics). All predictor variables were entered simultaneously into the regression model.

## Results

### Participant demographics

The demographics of web survey participants are presented in Table 1. We found a significant difference by race and income in our sample (p < 0.001), with a larger number of non-Hispanic black women in lower income categories than non-Hispanic white women. There were no other significant differences.

**Table 1:** **Respondent demographics**

	**Non-Hispanic black women (N = 404)**	**Non-Hispanic white women (N = 405)**	**P-value**
**Education**			0.73
High school or less	15% (n = 60)	17% (n = 68)	
Associate degree/some college	35% (n = 143)	35% (n = 143)	
College graduate or more	50% (n = 201)	48% (n = 194)	
**Income categories**			<0.001
Less than $25,000	28% (n = 111)	14% (n = 56)	
$25,000- $49,999	31% (n = 126)	35% (n = 142)	
$50,000-$99,999	29% (n = 115)	42% (n = 168)	
$100,000 or more	11% (n = 44)	8% (n = 31)	
Declined to answer	2% (n = 8)	2% (n = 8)	
**Pregnancy status**			0.97
Currently pregnant	41% (n = 164)	41% (n = 164)	
Planning pregnancy in next 12 months	59% (n = 240)	60% (n = 241)	

### Baseline behaviors associated with CMV risk

Kissing their child on the lips was the only behavior that was routinely performed by over half the women, and nearly one-third of women shared food with their children every day (Table 2). Nearly two-thirds of women (63.3%) reported engaging in at least one of the oral-exposure activities (sharing food, cup or utensils, kissing, putting pacifier in mouth) every day. Statistically significant differences by race were found for two survey items. Non-Hispanic white women were more likely to report *never or rarely washing their hands after changing a urine-only diaper* and *never or rarely washing their hands after wiping their child’s nose* compared to non-Hispanic black women.

**Table 2:** **Respondent baseline behaviors associated with exposures to CMV**

	**Non-Hispanic black women**	**Non-Hispanic white women**	P-value*
**Wash hands after changing poopy diaper**	3.0%	5.2%	0.08
Never or rarely	(n = 12)	(n = 21)	
**Wash hands after changing child’s (urine only)**	11.7%	18.5%*	<0.001
Never or rarely	(n = 47)	(n = 75)	
**Wash hands after wiping your child’s nose**	13.1%	27.4%*	<0.001
Never or rarely	(n = 53)	(n = 111)	
**Share food with child**	30.2%	30.1%	0.52
Every day	(n = 122)	(n = 122)	
**Share same cup with child**	15.6 %	15.3	0.49
Every day	(n = 63)	(n = 62)	
**Share eating utensils with child**	20.3%	22.0%	0.31
Every day	(n = 82)	(n = 89)	
**Kiss child on lips**	52.5%	54.6%	0.30
Every day	(n = 212)	(n = 221)	
**Put child’s pacifier in your mouth**	7.9%	7.2%	0.39
Every day	(n = 32)	(n = 29)	

*These represent p values for the overall distribution.

### CMV awareness and knowledge

#### Overall familiarity with CMV

**Figure 1:**
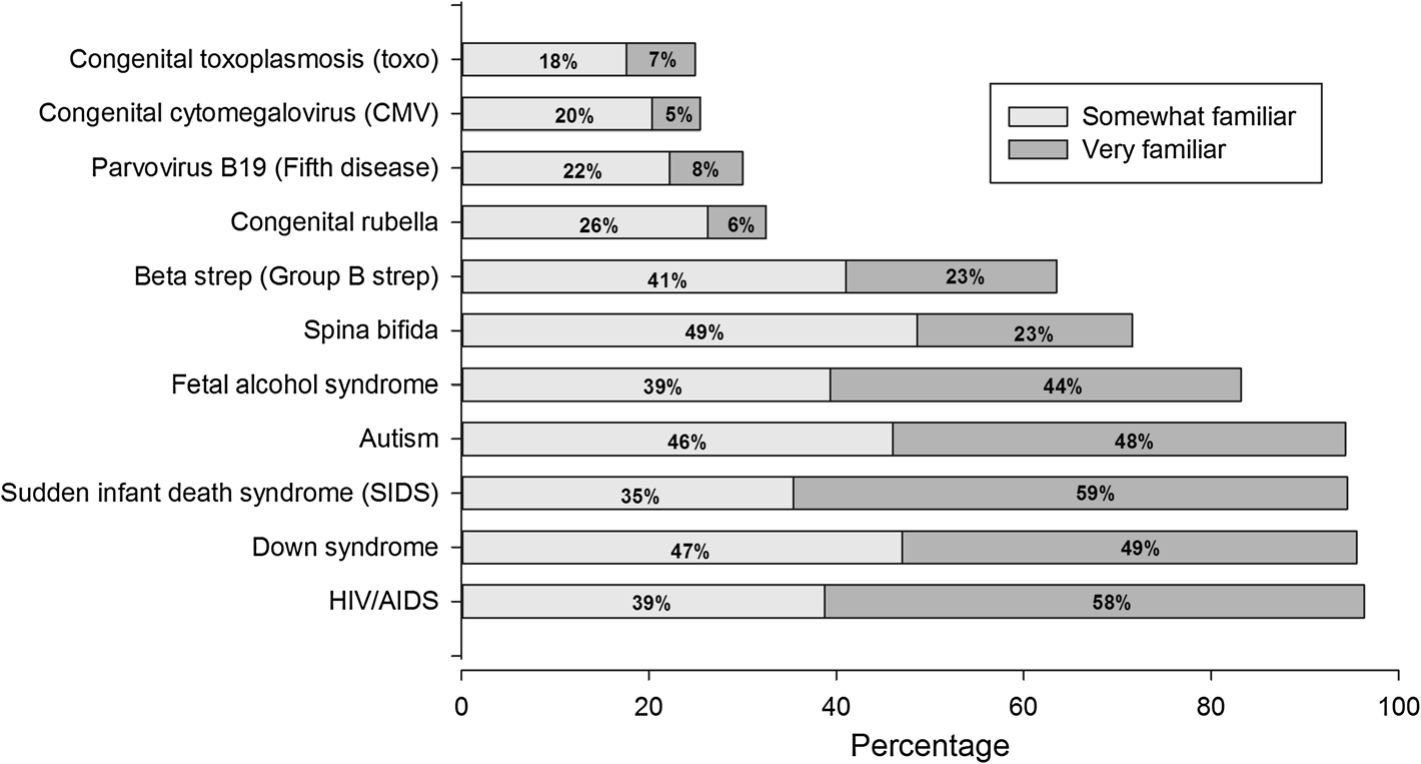
**Awareness of various disease conditions.** Women could answer “very familiar”, “somewhat familiar”, or “not at all familiar”. For easier viewing, only the percentages for the first two answer categories are plotted because the third, “not at all familiar”, is simply the percentage that will make the three categories sum to 100%.

Most women were unaware of CMV, with 75% indicating they were *not at all familiar with CMV* (Figure 1). Non-Hispanic black women were more likely to report they were *somewhat or very familiar with CMV* (30%) compared to non-Hispanic white women (21%, p < 0.001). Overall, women were more familiar with other health conditions affecting children than they were with CMV.

#### Comparison of pre and post knowledge measures

**Figure 2:**
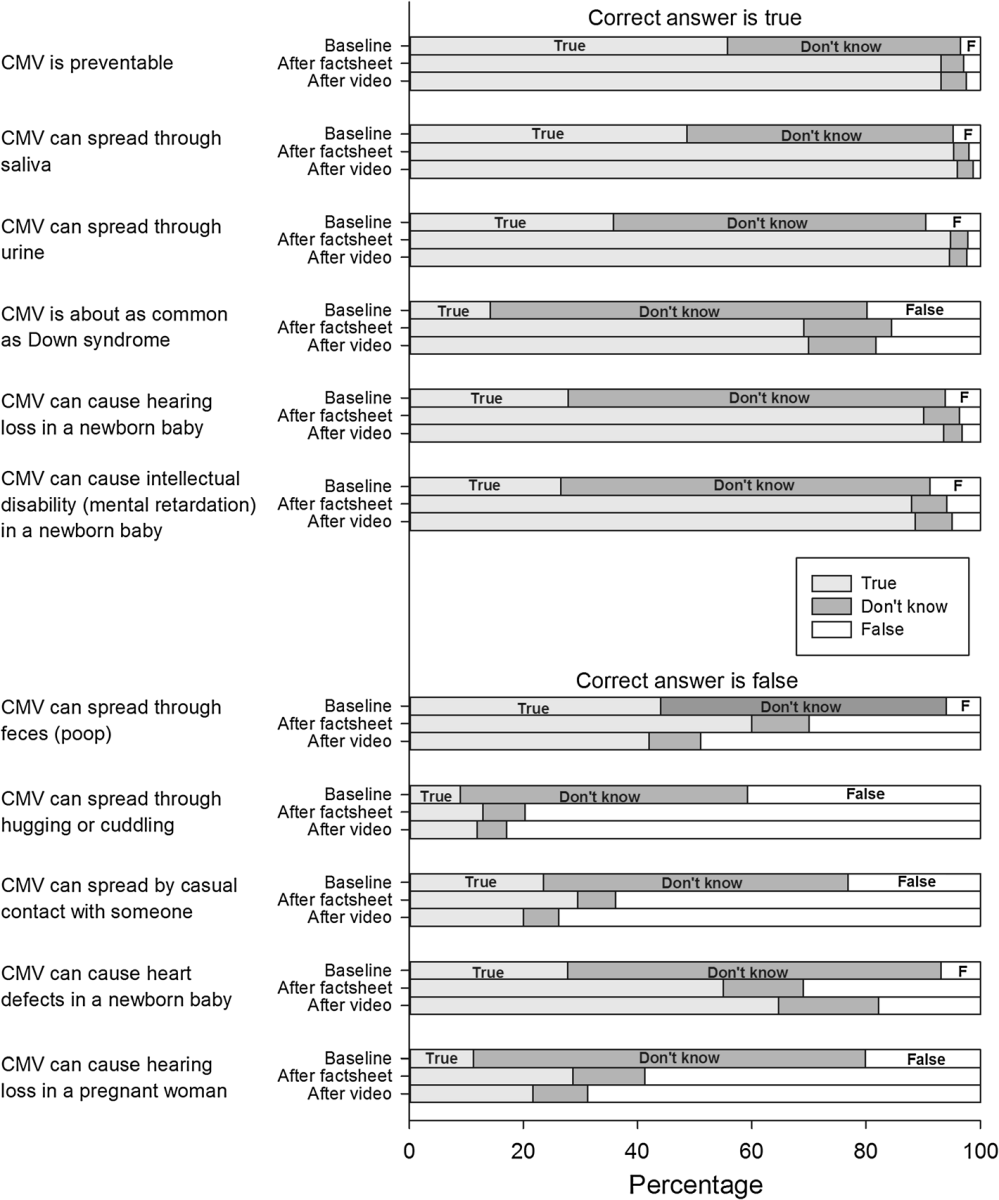
**Knowledge of CMV transmission and disease before and after viewing a factsheet or a video.** Statements that are true are in the top group; statements that are false are in the bottom group. Women could answer “true”, “false”, or “don’t know”. Although it is conceivable that CMV could be transmissible via feces, we treated this statement as false because CMV biology and epidemiology suggest that it is not a common transmission mode and it was not featured in the health education materials.

Following presentation of one of the health education materials, the proportion of women who correctly responded to CMV knowledge questions increased substantially and the proportion of women who did not know the correct response decreased substantially (Figure 2). Similarly, the summary post knowledge score (mean = 9.1, SD = 2.2) was significantly higher (p < 0.001) than the summary pre knowledge score (mean = 3.7, SD = 2.9). For the one multiple-choice question, at the pre knowledge measure, women responded that a pregnant woman is most likely to catch CMV from a young child (62%), a partner or spouse (18%), and a mosquito (20%). At the post knowledge measure, women responded that a pregnant woman is most likely to catch CMV from a young child (95%) (correct answer), a partner or spouse (4%), and a mosquito (1%).

#### Demographic differences in pre and post knowledge scores

In the analysis of variance for the pre knowledge score, we found a significant main effect for race, with non-Hispanic black women reporting a higher (p = 0.02) pre (mean = 3.9, SD = 2.9) knowledge score than non-Hispanic white women (mean = 3.4, SD = 2.9). There were no other significant findings for pre knowledge.

In the analysis of variance for the post knowledge score, we found significant main effects for race (p <0.001) and material type (p = 0.02), with non-Hispanic white women having a higher post knowledge score (mean = 9.3, SD = 2.0) compared to non-Hispanic black women (mean = 8.8, SD = 2.3). Similarly, women viewing the video also had higher post knowledge scores (mean = 9.2, SD = 2.1) compared to women viewing the factsheet (mean = 8.9, SD = 2.2). There were no interaction effects between race and material type. Having a higher pre knowledge score, having a college degree or more, and being pregnant were associated with a higher post knowledge score.

### Reactions to materials and messages

#### Materials appeal

**Figure 3:**
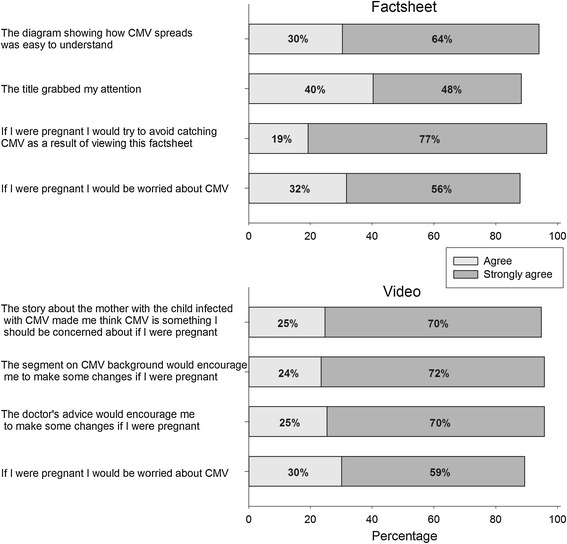
**Appeal of health education materials.** Women could answer “strongly disagree”, “disagree”, “agree”, or “strongly agree”. The percentages for the latter two categories are plotted.

Women responded very positively to each of the individual materials appeal questions (Figure 3). In the analysis of variance we found a significant main effect for race (p <0.001), with non-Hispanic black women having a higher appeal score (mean = 3.69, SD = 0.52) than non-Hispanic white women (mean = 3.50, SD = 0.58). There was also a main effect for materials type (p = 0.009), with those viewing the video having higher appeal scores (mean = 3.65, SD = 0.57) than those viewing the factsheet (mean = 3.55, SD = 0.53). There were no other significant findings. Overall, the average materials appeal score was high, with a mean of 3.6 (SD = 0.55) on a four-point scale.

#### Message involvement

The vast majority of women responded that after viewing the health education material they would be somewhat or very likely to look for information about CMV (90%) and talk about CMV with friends and family (89%). The average message involvement score was 2.48 (SD = .63), which was above the mid-point on a three-point scale. In the analysis of variance we found a significant main effect for race (p <0.001), with non-Hispanic black women having higher message involvement (mean = 2.64, SD = 0.54) than non-Hispanic white women (mean = 2.31, SD = 0.67). There was also a main effect for materials type (p <0.001), with those viewing the video having a slightly higher message involvement score (mean = 2.55, SD = 0.58) than those viewing the factsheet (mean = 2.41, SD = 0.66). There were no interaction effects between race and material type. Education was the only significant covariate in the model: having a college degree was associated with a lower message involvement score.

#### Support for CMV prevention behaviors

**Figure 4:**
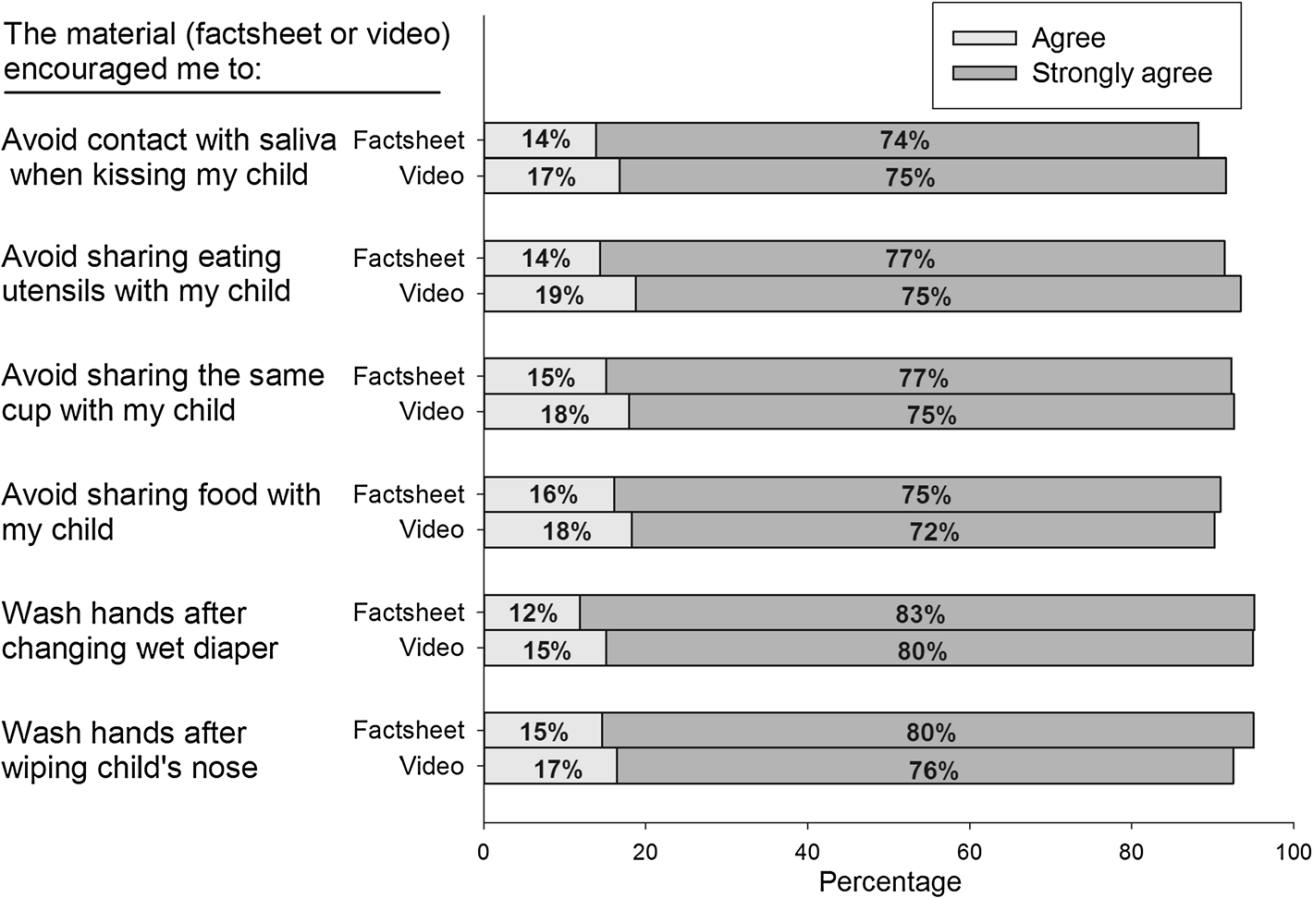
**Support for prevention behaviors after viewing health education materials.** Women could answer “strongly disagree”, “disagree”, “neither agree nor disagree”, “agree”, or “strongly agree”. The percentages for the latter two categories are plotted.

Women overwhelmingly agreed that the materials would encourage them to practice the individual prevention behaviors if they were pregnant (Figure 4). For these behaviors, we did not observe substantial differences in response to the factsheet versus the video.

#### Factors associated with support for CMV prevention behaviors

Increased support for CMV prevention behaviors was associated with viewing the video (vs. factsheet) and having higher scores for materials appeal, message involvement, post knowledge. The standardized coefficients for all predictors in the model are presented in Table 3. The overall model was also significant (p <0.001, adjusted *R*^2^ = 0.37).

**Table 3:** **Predictors for support for CMV prevention behaviors**

**Variable**	Standardized coefficient* (Beta)	**SD**	**P-value**	**Mean**
Materials appeal score (4 point scale)	0.50	0.55	<0.001	3.60
Message involvement score (3 point scale)	0.13	0.63	<0.001	2.47
Post knowledge score (12 point scale)	0.14	2.17	<0.001	9.05
Communication material (factsheet compared to video)	−0.08	0.50	0.006	1.50
Education (3 point scale)	0.06	0.73	0.06	2.33
Pregnancy status (pregnancy compared to planning)	0.02	0.49	0.54	1.59
Income (4 point scale)	0.01	0.91	0.81	2.34
Race (African-American compared to Caucasian)	0.01	0.50	0.83	1.50
Support for CMV Prevention Behaviors (5 point scale)		0.68		4.64

*A change in the standardized coefficient by the standard error of the regression parameter changes the dependent variable (support for CMV prevention behavior score) by 1 standard error of the dependent variable.

### Communication channels

**Figure 5:**
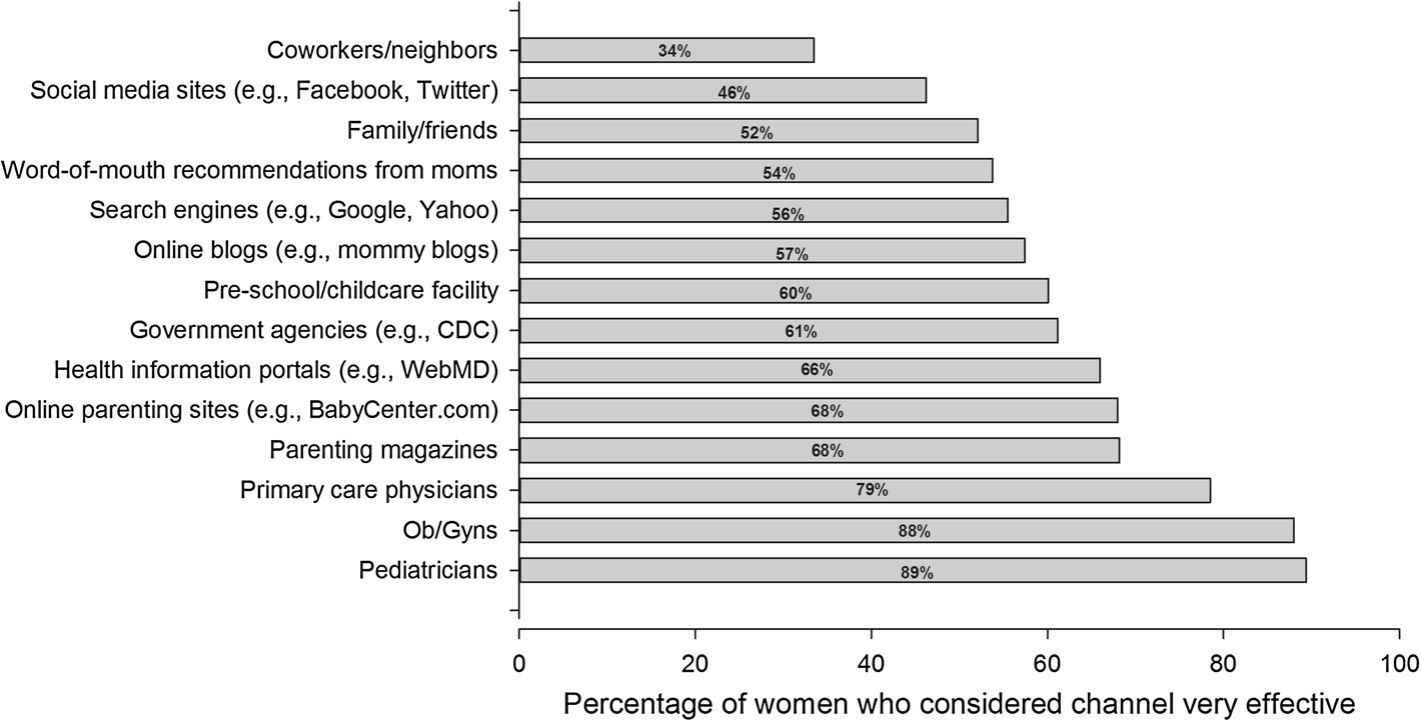
**Preferred channels for receiving CMV information.** Women could answer “not at all effective”, “somewhat effective”, or “very effective”. The percentages for the last category are plotted.

Women indicated (Figure 5) that the most effective ways for educating mothers about CMV were through physicians (e.g., pediatricians) and channels where they could search on their own for CMV information (e.g., magazines and websites). Direct communication with people aside from physicians (such as friends, family, and other moms) was less preferable as a source of CMV information.

## Discussion

Among the women we surveyed, viewing either the educational factsheet or video substantially increased knowledge of CMV and prevention behaviors, and was associated with very strong support for prevention behaviors during pregnancy. Furthermore, the women found the materials to be highly appealing and indicated that they would try to avoid catching CMV if they were pregnant.

There was some evidence the video may have been slightly more effective than the factsheet. The broader health communication literature provides some evidence that video formats can be more effective than written formats [26], particularly among low literacy populations [27]. Some of the difference may be attributable to the use of personal narrative (i.e., mother telling the story of her child with CMV), which can improve message recall [28,29]. However, the difference in effectiveness was too small to be able to draw definitive conclusions; the larger finding was that both materials were highly effective in increasing knowledge and influencing attitudes.

Our findings also indicate that non-Hispanic black women found both health education materials slightly more appealing than non-Hispanic white women. Other studies have found that similarity between minority actors and viewers can result in greater message recall and favorable attitudes among minority respondents [30].

Both non-Hispanic black and white women increased their knowledge scores significantly from pre to post (non-Hispanic black women: 3.9 to 8.8; non-Hispanic white women: 3.4 to 9.3). Interestingly, the pre knowledge score was higher among non-Hispanic black women while the post knowledge score was higher among non-Hispanic white women. Because we controlled for pre knowledge score, our findings suggest the effect of the materials may have been slightly more pronounced on non-Hispanic white women, but once again, the racial differences were small compared to the overall increases in knowledge.

Findings from the regression model provide additional insights for encouraging CMV prevention behaviors among women. Significant predictors for encouraging support for CMV prevention behaviors included positive reactions to materials and messages, and higher post knowledge scores. Viewing the video (versus factsheet) was also a significant predictor, but less important than the other predictors. Interestingly, none of the demographic variables (race, education, pregnancy status or income) were significant predictors for encouraging CMV prevention behaviors. Overall these findings suggest that the keys to developing health education materials that encourage CMV prevention behaviors include using strategies that 1) appeal to and involve the target audiences (e.g., personal narratives; images and actors reflective of the target population(s)); and 2) give the audience new information (in this instance, increasing their knowledge about CMV).

There are important limitations to note regarding this study. We used a convenience sample, thus findings from this study cannot be generalized to the general population of non-Hispanic black and white mothers. In addition, as with all self-reported data, there is the possibility of social desirability bias in responses to survey questions. Similarly, while findings from the survey suggest that health education materials may encourage the adoption of prevention behaviors, we had no direct behavioral measures in the study.

It is important to note that post material presentation measures, including appeal, knowledge of CMV, and motivation to engage in CMV prevention behaviors were assessed immediately following the presentation of the health education material. If these had been measured at later time points, the impact might have been diminished.

## Conclusions

Overall, we found that health education materials can improve women’s knowledge and awareness of CMV and their motivation to practice CMV prevention behaviors. Given the low prevalence of CMV awareness among U.S. women currently, these findings are encouraging and suggest that appropriate dissemination of CMV health education materials can help raise awareness among target audiences. Additionally, there is some indication that these materials may also encourage women to engage in behaviors that will reduce their exposures to CMV during pregnancy. The potential impact of these health education materials requires further exploration, especially regarding the extent to which viewing the materials can lead to behavior changes and reductions in the risk of acquiring CMV infection.

## Additional file

Additional file 1:
**Content of web-based survey.**

## Abbreviations

CDC: Centers for disease control and prevention
CMV: Cytomegalovirus
cCMV: Congenital cytomegalovirus
