# Supplementary material for: Educating women about congenital cytomegalovirus: assessment of health education materials through a web-based survey
Source: BMC Womens Health. 2014 Nov 30;14:144. doi: 10.1186/s12905-014-0144-3 (PMC4260245; doi:10.1186/s12905-014-0144-3)
Supplement: Additional file 1: — Content of web-based survey. [file 12905_2014_144_MOESM1_ESM.docx]

CMV Web Survey^[[1]](#footnote-1)^

**INTRODUCTION**

Thank you for agreeing to participate in the survey. This survey is being conducted on behalf of the Centers for Disease Control and Prevention (CDC). Your participation is important and will help CDC in their efforts to educate parents about preventing birth defects.

The survey will ask about your interactions with your youngest child, knowledge and attitudes about birth defects, and your opinions on draft educational materials to help parents prevent birth defects.

Please answer questions as honestly as possible. There is no right or wrong answer and all your responses will be confidential. This is a voluntary study and you can stop at any time. The survey will take approximately 10-15 minutes.

If you’re ready to begin, please select the forward arrow at the bottom of your screen.

**I. Baseline Awareness/Knowledge of CMV**

[*Within each question on the survey, the order of the sub-questions was randomized*]

1. When your youngest child was in diapers, how often did you wash your hands or use hand sanitizer after each of the following? (If your youngest child is still in diapers, think about what you currently do.)

|  | Never | Rarely | Some of the time | Most of the time | Always |
| --- | --- | --- | --- | --- | --- |
| 1. After changing your child’s dirty (poopy) diaper | 1 | 2 | 3 | 4 | 5 |
| 1. After changing your child’s wet (urine only) diaper | 1 | 2 | 3 | 4 | 5 |
| 1. After wiping your child’s nose | 1 | 2 | 3 | 4 | 5 |

1. When your youngest child was in diapers, how often did you do each of the following? (If your youngest child is still in diapers, think about what you currently do.)

|  | Never | Rarely | 1-2 days a week | 3-5 days a week | Every day |
| --- | --- | --- | --- | --- | --- |
| 1. Share food with your child (take bites from the same food) | 1 | 2 | 3 | 4 | 5 |
| 1. Share the same cup with your child | 1 | 2 | 3 | 4 | 5 |
| 1. Share eating utensils (fork or spoon) with your child | 1 | 2 | 3 | 4 | 5 |
| 1. Kiss your child on their lips | 1 | 2 | 3 | 4 | 5 |
| 1. Put a pacifier in your mouth after it has been in your child’s mouth | 1 | 2 | 3 | 4 | 5 |

The following questions are about an infection called cytomegalovirus also known as CMV.

1. A pregnant woman is most likely to catch CMV from which of the following:
   1. Young child
   2. Partner or spouse
   3. Mosquito
2. Please indicate if you think the following statements are true or false.

|  | True | False | Don’t Know |
| --- | --- | --- | --- |
| 1. CMV can cause intellectual disability (mental retardation) in a newborn baby | 1 | 2 | 3 |
| 1. CMV can cause hearing loss in a pregnant woman | 1 | 2 | 3 |
| 1. CMV can spread through urine | 1 | 2 | 3 |
| 1. CMV can spread through saliva | 1 | 2 | 3 |
| 1. CMV can cause heart defects in a newborn baby | 1 | 2 | 3 |
| 1. CMV can spread by casual contact with someone | 1 | 2 | 3 |
| 1. CMV can cause hearing loss in a newborn baby | 1 | 2 | 3 |
| 1. CMV is about as common as Down Syndrome | 1 | 2 | 3 |
| 1. CMV can spread through feces (poop) | 1 | 2 | 3 |
| 1. CMV is preventable | 1 | 2 | 3 |
| 1. CMV can spread through hugging or cuddling | 1 | 2 | 3 |

1. Please indicate how familiar you are with each of the following health conditions (where 1=very familiar and 3=not at all familiar).

|  | Very familiar | Somewhat familiar | Not at all familiar |
| --- | --- | --- | --- |
| 1. Congenital rubella syndrome | 1 | 2 | 3 |
| 1. Beta strep (Group B strep) | 1 | 2 | 3 |
| 1. HIV/AIDS | 1 | 2 | 3 |
| 1. Congenital cytomegalovirus (CMV) | 1 | 2 | 3 |
| 1. Down syndrome | 1 | 2 | 3 |
| 1. Sudden infant death syndrome (SIDS) | 1 | 2 | 3 |
| 1. Fetal alcohol syndrome | 1 | 2 | 3 |
| 1. Autism | 1 | 2 | 3 |
| 1. Spina bifida | 1 | 2 | 3 |
| 1. Congenital toxoplasmosis (toxo) | 1 | 2 | 3 |
| 1. Parvovirus B19 (Fifth disease) | 1 | 2 | 3 |

*Programming Note:*

*Respondents were split here as follows.*

*Path 1: Half of the respondents were randomly assigned to Section II, followed by Section IV (skipped Section III).*

*Path 2: Half of the respondents were randomly assigned to Section III, followed by Section IV (skipped Section II).*

**II. CMV FACTSHEET: Message Appeal and Understanding**

Next, you’ll be presented with a factsheet about CMV that you might see at a doctor’s office or on the web. You’ll be asked a few questions after viewing it.

Please click the forward arrow to continue.

Please take a few minutes to read the factsheet and then click the forward arrow to continue.

[CMV Material 1: EMBED FACTSHEET]

1. Were you able to view the factsheet?
2. Yes
3. No THANK AND TERMINATE
4. In the next week or so, how likely are you to do the following (where 1=very likely and 3=not at all likely):

|  | Very likely | Somewhat likely | Not at all likely |
| --- | --- | --- | --- |
| 1. Talk to your friends/family about CMV | 1 | 2 | 3 |
| 1. Look for more information about CMV (e.g., talk to your doctor or search the Internet) | 1 | 2 | 3 |

1. Please indicate how much you agree or disagree with the following statements (where 1=strongly agree and 5=strongly disagree).

| The factsheet encouraged me to… | Strongly Agree | Agree | Neither agree nor disagree | Disagree | Strongly disagree |
| --- | --- | --- | --- | --- | --- |
| 1. Wash my hands after wiping my child’s nose if I were pregnant | 1 | 2 | 3 | 4 | 5 |
| 1. Wash my hands after changing my child’s wet (urine only) diaper if I were pregnant | 1 | 2 | 3 | 4 | 5 |
| 1. Avoid sharing food with my child (take bites from the same food) if I were pregnant | 1 | 2 | 3 | 4 | 5 |
| 1. Avoid sharing the same cup with my child if I were pregnant | 1 | 2 | 3 | 4 | 5 |
| 1. Avoid sharing eating utensils (fork or spoon) with my child if I were pregnant | 1 | 2 | 3 | 4 | 5 |
| 1. Avoid contact with saliva when kissing my child (e.g. by kissing on the cheek or forehead) if I were pregnant | 1 | 2 | 3 | 4 | 5 |

1. A pregnant woman is most likely to catch CMV from which of the following:

1. Young child

2. Partner or spouse

3. Mosquito

1. Thinking about the factsheet you just read, please indicate how much you agree or disagree with each of the following statements (where 1=strongly agree and 4=strongly disagree).

|  | Strongly Agree | Agree | Disagree | Strongly Disagree |
| --- | --- | --- | --- | --- |
| 1. I thought the diagram in the upper right corner showing how CMV spreads from a young child to a pregnant woman was easy to understand. | 1 | 2 | 3 | 4 |
| 1. The title on the factsheet grabbed my attention. | 1 | 2 | 3 | 4 |
| 1. If I were pregnant I would try to avoid catching CMV as a result of viewing this factsheet | 1 | 2 | 3 | 4 |
| 1. If I were pregnant I would be worried about CMV | 1 | 2 | 3 | 4 |

1. Please indicate if you think the following statements are true or false.

|  | True | False | Don’t Know |
| --- | --- | --- | --- |
| 1. CMV can cause intellectual disability (mental retardation) in a newborn baby | 1 | 2 | 3 |
| 1. CMV can cause hearing loss in a pregnant woman | 1 | 2 | 3 |
| 1. CMV can spread through urine | 1 | 2 | 3 |
| 1. CMV can spread through saliva | 1 | 2 | 3 |
| 1. CMV can cause heart defects in a newborn baby | 1 | 2 | 3 |
| 1. CMV can spread by casual contact with someone | 1 | 2 | 3 |
| 1. CMV can cause hearing loss in a newborn baby | 1 | 2 | 3 |
| 1. CMV is about as common as Down Syndrome | 1 | 2 | 3 |
| 1. CMV is preventable | 1 | 2 | 3 |
| 1. CMV can spread through hugging or cuddling | 1 | 2 | 3 |
| 1. CMV can spread through feces (poop) | 1 | 2 | 3 |

**III. CMV VIDEO: Message Appeal and Understanding**

Next, you’ll be presented with a video about CMV that you might see in a doctor’s office or on the web. You’ll be asked a few questions after viewing it.

Click the forward arrow to proceed when you are ready.

CMV Material 2: [EMBED VIDEO]

Once you have viewed the video please click on the forward arrow below to continue with the survey.

1. Were you able to view and hear the video?
2. Yes
3. No THANK AND TERMINATE
4. In the next week or so, how likely are you to do the following (where 1=very likely and 3=not at all likely):

|  | Very likely | Somewhat likely | Not at all likely |
| --- | --- | --- | --- |
| 1. Talk to your friends/family about CMV | 1 | 2 | 3 |
| 1. Look for more information about CMV (e.g., talk to your doctor or search the Internet) | 1 | 2 | 3 |

1. Please indicate how much you agree or disagree with the following statements (where 1=strongly agree and 5=strongly disagree).

| The video encouraged me to… | Strongly agree | Agree | Neither agree nor disagree | Disagree | Strongly disagree |
| --- | --- | --- | --- | --- | --- |
| 1. Wash my hands after wiping my child’s nose if I were pregnant | 1 | 2 | 3 | 4 | 5 |
| 1. Wash my hands after changing my child’s wet (urine only) diaper if I were pregnant | 1 | 2 | 3 | 4 | 5 |
| 1. Avoid sharing food with my child (take bites from the same food) if I were pregnant | 1 | 2 | 3 | 4 | 5 |
| 1. Avoid sharing the same cup with my child if I were pregnant | 1 | 2 | 3 | 4 | 5 |
| 1. Avoid sharing eating utensils (fork or spoon) with my child if I were pregnant | 1 | 2 | 3 | 4 | 5 |
| 1. Avoid contact with saliva when kissing my child (e.g. by kissing on the cheek or forehead) if I were pregnant | 1 | 2 | 3 | 4 | 5 |

1. A pregnant woman is most likely to catch CMV from which of the following:

1. Young child

2. Partner or spouse

3. Mosquito

1. Thinking about the video you just saw, please indicate how much you agree or disagree with each of the following statements (where 1=strongly agree and 4=strongly disagree).

|  | Strongly Agree | Agree | Disagree | Strongly Disagree |
| --- | --- | --- | --- | --- |
| 1. The story about the mother with the child infected with CMV made me think CMV is something I should be concerned about if I were pregnant. | 1 | 2 | 3 | 4 |
| 1. The message from the CDC in the video would encourage me to make some changes if I were pregnant. | 1 | 2 | 3 | 4 |
| 1. The doctor’s advice in the video would encourage me to make some changes if I were pregnant. | 1 | 2 | 3 | 4 |
| 1. If I were pregnant I would be worried about CMV | 1 | 2 | 3 | 4 |

1. Please indicate if you think the following statements are true or false.

|  | True | False | Don’t Know |
| --- | --- | --- | --- |
| 1. CMV can cause intellectual disability (mental retardation) in a newborn baby | 1 | 2 | 3 |
| 1. CMV can cause hearing loss in a pregnant woman | 1 | 2 | 3 |
| 1. CMV can spread through urine | 1 | 2 | 3 |
| 1. CMV can spread through saliva | 1 | 2 | 3 |
| 1. CMV can cause heart defects in a newborn baby | 1 | 2 | 3 |
| 1. CMV can spread by casual contact with someone | 1 | 2 | 3 |
| 1. CMV can cause hearing loss in a newborn baby | 1 | 2 | 3 |
| 1. CMV is about as common as Down Syndrome | 1 | 2 | 3 |
| 1. CMV is preventable | 1 | 2 | 3 |
| 1. CMV can spread through hugging or cuddling | 1 | 2 | 3 |
| 1. CMV can spread through feces (poop) | 1 | 2 | 3 |

**IV. CMV Information Sources/Channels**

The following questions ask about where you might expect to get information about CMV.

1. What do you think might be the best way or most effective channel for educating mothers about CMV?  Please rate how effective each of following channels would be in getting CMV information to mothers like you.

Would you say they are very effective, somewhat effective, or not at all effective…

|  | Very effective | Somewhat effective | Not at all effective |
| --- | --- | --- | --- |
| 1. Pre-school/child care facility | 1 | 2 | 3 |
| 1. Family/friends | 1 | 2 | 3 |
| 1. Coworkers/neighbors | 1 | 2 | 3 |
| 1. Doctor: OB-GYN | 1 | 2 | 3 |
| 1. Doctor: Pediatrician | 1 | 2 | 3 |
| 1. Doctor: Primary Care Physician | 1 | 2 | 3 |
| 1. Online: Health information portals (like WebMD) | 1 | 2 | 3 |
| 1. Online: Search engines (like Google, Yahoo) | 1 | 2 | 3 |
| 1. Online: Media sites (like BabyCenter.com, ParentsPlace.com) | 1 | 2 | 3 |
| 1. Online: Social media sites (like Facebook, Twitter, YouTube) | 1 | 2 | 3 |
| 1. Online: Blogs (like Mommy Blogs) | 1 | 2 | 3 |
| 1. Parenting magazines | 1 | 2 | 3 |
| 1. Word-of-mouth recommendations from other mothers | 1 | 2 | 3 |
| 1. Government agencies (like CDC) | 1 | 2 | 3 |
| 1. Other | 1 | 2 | 3 |

If Q18(Other) = 1 or 2, GO TO Q18a. ELSE, GO TO THANK.

18a. In the previous question you marked some “Other” way as effective. What other channel do you consider effective in getting CMV information to mothers like you?

WRITE IN YOUR RESPONSE_______________________________________

THANK: Thank you for taking the time to answer these questions. Have a nice day.

1. Demographic information was obtained separately by Harris Interactive, the survey administrators. [↑](#footnote-ref-1)
